# TRPC1 expression and function inhibit ER stress and cell death in salivary gland cells

**DOI:** 10.1096/fba.1021

**Published:** 2018-11-16

**Authors:** Pramod Sukumaran, Yuyang Sun, Fredice Quenum Zangbede, Viviane Nascimento da Conceicao, Bibhuti Mishra, Brij B. Singh

**Affiliations:** ^1^ Department of Periodontics, School of Dentistry University of Texas Health Science Center San Antonio Texas; ^2^ Department of Biomedical Sciences, School of Medicine and Health Sciences University of North Dakota Grand Forks North Dakota

**Keywords:** ER stress, salivary gland, SOCE, TRPC1, Tunicamycin

## Abstract

Disturbances in endoplasmic reticulum (ER) Ca^2+^ homeostasis have been associated with many diseases including loss of salivary glands. Although significant progress has been accomplished which led to the increase in our understanding of the cellular responses to ER stress, the factors/ion channels that could inhibit ER stress are not yet identified. Here, we show that TRPC1 (transient receptor potential canonical 1) is involved in regulating Ca^2+^ homeostasis and loss of TRPC1 decreased ER Ca^2+^ levels, inhibited the unfolded protein response (UPR), that induced loss of salivary gland cells. We provide further evidence that ER stress‐inducing agents (Tunicamycin [Tu] and Brefeldin A [BFA]) disrupt Ca^2+^ homeostasis by directly inhibiting TRPC1‐mediated Ca^2+^ entry, which led to ER stress in salivary gland cells. Moreover, induction of ER stress lead to an increase in C/EBP homologous protein (CHOP) expression, which decreased TRPC1 expression and subsequently attenuated autophagy along with increased apoptosis. Importantly, TRPC1^−/−^ mice showed increased ER stress, increased immune cell infiltration, loss of Ca^2+^ homeostasis, decreased saliva secretion, and decreased salivary gland survival. Finally, restoration of TRPC1 not only maintained Ca^2+^ homeostasis but also inhibited ER stress that induced cell survival. Overall these results suggest a significant role of TRPC1 Ca^2+^ channels in ER stress and homeostatic function/survival of salivary gland cells.

**Abbreviations**
[Ca^2+^]_i_: intracellular‐free calcium concentration or cytoplasmic‐free calcium concentration
ER: endoplasmic reticulum
HBSS: Hank’s balanced salt solution
ORAI: calcium release‐activated calcium channel protein
PBS: phosphate‐buffered saline
PM: plasma membrane
SERCA: sacro/Endoplasmic Reticulum Ca^2+^‐ATPase
SOCE: store‐operated calcium entry
SS: Sjögren’s syndrome
STIM: stromal interaction molecule
Tg: thapsigargin
TRPC: transient receptor potential canonical channel
UPR: unfolded protein response

## 1 INTRODUCTION

Calcium is a ubiquitous second messenger that modulates most of the cellular functions including gene expression and cellular homeostasis,^1^, ^2^ neurotransmitter release and neuronal function,^3^, ^4^ and modulation of metabolism and cell survival.^5^ The known molecular regulators of cell calcium homeostasis, such as calcium release‐activated calcium channel (ORAI), stromal interaction molecule 1 (STIM1) and TRPC channels are all implicated in modulating Ca^2+^ entry in both excitable and nonexcitable cells. Importantly, TRPC and ORAI channels have been suggested as components of Ca^2+^ influx channels that are activated in response to agonist‐mediated Ca^2+^‐signaling cascades and/or store depletion. Activation of the G‐protein‐coupled receptors leads to the activation heterotrimeric G‐protein (G_q/11_) which hydrolysis PIP_2_ that generates two second messengers, IP_3_ and DAG. IP_3_ binds to the IP_3_R and initiates Ca^2+^ release from the internal ER stores, which allows STIM1 to rearrange in order to activate plasma membrane Ca^2+^ influx channels mainly ORAIs and TRPCs. Ca^2+^ entry from these channels are essential for refilling of the ER Ca^2+^ stores as well as in regulating cellular functions. Similarly, mitochondrial, lysosomal, and nuclear Ca^2+^ levels are also regulated by Ca^2+^ permeable ion channels localized on the plasma membrane^6^ that modulates cellular functions. Thus, loss of cellular Ca^2+^ homeostasis especially upon inhibition of Ca^2+^ entry disrupts Ca^2+^ signaling in the cell, inducing response that promotes cell demise.

Ca^2+^ is a major player in the regulation of cell death, both at the early and late stages of apoptosis, and severe Ca^2+^ dysregulation can induce ER stress‐mediated apoptosis in response to various pathological conditions.^7^, ^8^, ^9^, ^10^ Apoptosis is a controlled cellular process that is characterized by distinctive changes such as cellular shrinkage, cytoplasmic blebbing, and condensation of chromatin, which is initiated by activation of caspases and upregulation of pro‐apoptotic proteins that are also modulated by intracellular Ca^2+^ levels.^11^, ^12^, ^13^, ^14^ Salivary gland cells are susceptible to ER stress related to their secretory activity and the complexity of synthesized secretory products.^15^ Studies have shown that ER stress is activated in minor salivary gland epithelial cells from Sjögren's syndrome (SS) patients. Moreover, an interplay between ER stress‐induced autophagy and apoptosis has also been suggested with regard to SS autoantigens Ro/SSA and La/SSB.^13^ The ER is an important intracellular organelle that is not only important for regulating Ca^2+^ homeostasis but is also essential for the synthesis and folding of proteins. The presence of cellular stressors initiates a signaling cascade that induces the unfolded protein response (UPR) that is critical for the reestablishing of the cellular homeostasis. Three signaling pathways that are initiated by the kinases IRE1, PERK, and the transcription factor ATF6 have been identified during UPR activation.^9^ These three pathways coordinate the cellular response to unfolded proteins, which include (a) downregulation of protein translation; (b) enhanced expression of ER chaperone proteins that promote protein refolding; and (c) activation of proteases involved in the degradation of misfolded proteins. Importantly, ER‐resident kinase PERK is the most essential for ER stress, which when activated phosphorylates the translational initiation factor eIF2α that modulates the ER stress response. In addition, if the UPR is not able to restore cellular homeostasis, autophagy is induced leading to degradation of organelles and proteins necessary for their survival. Conversely, prolonged or severe ER stress can lead to the activation of apoptotic cell death, which requires the ATF4‐dependent transcription factor C/EBP homologous protein (CHOP).^8^ However, although it is apparent that ER stress plays a major role in cell death, molecular factors that induce ER stress are still not known. Hence, a better understanding of the molecular mechanism(s) which induces ER stress could aid in preventing the ER stress‐induced cell death.

Ca^2+^ influx followed by ER store depletion accomplishes several critical cellular functions. First, this Ca^2+^ influx replenishes the ER Ca^2+^ stores, thereby maintaining its ability to release Ca^2+^ upon subsequent stimuli. Moreover, this is also critical since Ca^2+^ concentrations within the ER must be maintained at sufficient levels for the organelle to carry out many of its fundamental functions.^8^, ^9^, ^10^ Previous studies from our laboratory and others have shown that transient receptor potential canonical channel‐1 (TRPC1) is involved in regulating Ca^2+^ homeostasis in salivary gland cells.^16^ Interestingly, in human salivary epithelial cell lines, TRPC1 has shown to be the major Ca^2+^ influx channel and knockdown of TRPC1 inhibited salivary gland function; however, its role in ER stress is not yet established. In the present study, we have examined the role TRPC1 in ER stress. Our results clearly show that blocking TRPC1 activity or loss of TRPC1 expression inhibited Ca^2+^ homeostasis that lead to ER stress. Furthermore, our data provided the mechanism where ER stress‐induced expression of CHOP modulates TRPC1 expression, which further inhibits Ca^2+^ homeostasis and inhibits autophagy that increases apoptosis in human salivary cells.

## 2 EXPERIMENTAL PROCEDURES

### 2.1 Cell culture reagents and overexpression of TRPC1

Human salivary gland (HSG) cells were cultured in their respective medium along with various supplements as previously described.^17^, ^18^ Cells were maintained at 37°C with 95% humidified air and 5% CO_2_ and were passaged as needed. Culture medium was changed twice weekly and cells were maintained in complete media until reaching 90% confluence. Cells were transfected with individual siRNA (50 nM) and/or plasmids using Lipofectamine 2000 in the Opti‐MEM medium as per supplier's instructions (Invitrogen) and assayed after 48 hours. Antibodies that were used in this study are described in the figures. All other reagents used were of molecular biology grade obtained from Sigma chemicals unless mentioned otherwise.

### 2.2 Cell viability assays

Cells were seeded in 96‐well plates at a density of 0.5×10^5^ cells/well. The cultures were grown for 24 hours followed by addition of fresh medium prior to the experiment. Cell viability was measured by using the MTT method. 20 μl of MTT reagent (5 mg/ml MTT in PBS) was added to each well and incubated in a CO_2_ incubator for 4 hours. The resulting formazan dye was extracted with 100 μl of 0.01 N HCl in isopropanol and the absorbance was measured in a microplate reader (Molecular Device, Sunnyvale, CA) at 570 and 650 nm. Cell viability was expressed as a percentage of the control culture.

### 2.3 Electrophysiology

For patch clamp experiments, coverslips with cells were transferred to the recording chamber and perfused with an external Ringer's solution of the following composition (mM): NaCl, 145; CsCl, 5; MgCl2, 1; CaCl2, 1; Hepes, 10; Glucose, 10; pH 7.3 (NaOH). Whole‐cell currents were recorded using an Axopatch 200B (Axon Instruments, Inc., Union City, CA). The patch pipette had resistances between 3 and 5 M after filling with the standard intracellular solution that contained the following (mM): cesium methane sulfonate, 150; NaCl, 8; Hepes, 10; EGTA, 10; pH 7.2 (CsOH). With a holding potential 0 mV, voltage ramps ranging from −100 mV to +100 mV and 100 milliseconds duration were delivered at 2 seconds intervals after whole‐cell configuration was formed. Currents were recorded at 2 kHz and digitized at 5‐8 kHz. pClamp 10.1 software was used for data acquisition and analysis. Basal leak was subtracted from the final currents and average currents are shown. All experiments were carried out under room temperature.

### 2.4 Calcium measurement

Cells were incubated with 2 μM fura‐2 (Molecular Probes) for 45 minutes, washed twice with Ca^2+^‐free SES (standard external solution, include 10 mM HEPES, 120 mM NaCl, 5.4 mM KCl, 1 mM MgCl_2_, 10 mM glucose, pH 7.4) buffer. For fluorescence measurements, the fluorescence intensity of Fura‐2‐loaded control cells was monitored with a CCD camera‐based imaging system (Compix Inc., Cranberry, PA) mounted on an Olympus XL70‐inverted microscope equipped with an Olympus 40× (1.3 NA) objective. A monochrome dual wavelength enabled alternative excitation at 340 and 380 nm, whereas the emission fluorescence was monitored at 510 nm with an Orca imaging camera (Hamamatsu Photonics K.K., Hamamatsu, Japan). The images of multiple cells collected at each excitation wavelength were processed using the C imaging, PCI software (Compix Inc.), to provide ratios of Fura‐2 fluorescence from excitation at 340 nm to that from excitation at 380 nm (F340/F380). Fluorescence traces shown represent [Ca^2+^]_i_ values that are averages from at least 30‐40 cells and are a representative of results obtained in at least three to four individual experiments.^17^, ^19^

### 2.5 Membrane preparations and western blot analyses

Cells were harvested and stored at −80°C. Crude lysates were prepared from HSG cells as described previously.^20^ Protein concentrations were determined, using the Bradford reagent (Bio‐Rad), and 25‐50 µg of proteins were resolved on 4%‐12% SDS‐Bis‐Tris gels transferred to PVDF membranes and probed with respective antibodies. Peroxidase‐conjugated respective secondary antibodies were used to label the proteins. The proteins were detected by enhanced chemiluminescence detection kit (SuperSignal West Pico; Pierce). All the information about the antibody used is given in the Supplemental Table S1. Densitometric analysis was performed using ImageJ analysis and results were corrected for protein loading by normalization for β‐actin (Cell Signaling, MA) expression as described.^2^, ^20^, ^21^, ^22^

### 2.6 Caspase 3 activity

Caspase 3 activity was measured using abcam Caspase 3 assay kit (Abcam Plc, MA). One million cells were isolated using cell lysis buffer and the liquid fraction was used to analysis the caspase 3 activity as manufacturer's instructions. The assay is based on spectrophotometric detection of the chromophore p‐nitroaniline (p‐NA) after cleavage from the labeled substrate DEVD‐pNA. The sample absorbance was measured at 405 nm and graph was plotted using absorbance value.^19^

### 2.7 Animal and saliva secretion

Eight to ten months old male C57BL/6 control and TRPC1 KO mice were used for these experiments. All animals were housed in a temperature controlled room under a 12/12‐hour light/dark cycle with ad libitum access to food and water, and experiments were carried out as per the institutional guidelines for the use and care of animals. For saliva measurement, mice were anesthetized, whole saliva was collected after stimulation with 0.5 mg of pilocarpine/kg body weight was measured as previously described.^23^, ^24^

### 2.8 Histological and immunofluorescence analysis

For histological analysis, animals (WT and TRPC1 KO mice) were perfused and salivary glands were quickly dissected out, embedded in optimal cutting temperature (OCT), and snap frozen. Serial horizontal cryosections of 10 μm in thickness were placed on silane prep slides (Sigma‐Aldrich, St. Louis, MO). The slides were air‐dried overnight and fixed in fresh acetone for 10 seconds at room temperature. Acetone‐fixed sections were processed immediately for hematoxylin and eosin (H&E). For immunofluorescence staining, the frozen salivary gland tissue sections were first stained for the detection of caspase‐3, using an affinity‐purified anti‐mCaspase‐3 Rabbit IgG (Cell Signaling, Danvers, MA) and detected with Alexa Fluor^®^ 546 Goat anti‐Rabbit IgG (Life Technologies, Grand Island, NY). The staining was sequentially repeated for Aquaporin 5 using an affinity‐purified Rabbit anti‐mAquaporin 5 (AQP5) (Abcam, Cambridge, MA) and visualized with Alexa Fluor^®^ 488 Goat anti‐Rabbit IgG (Life Technologies, Grand Island, NY) each additional staining. The sections were mounted using FluorSave reagent (Calbiochem, La Jolla, CA) containing 0.3 μM 4′, 6′‐diamidino‐2‐phenylindole (DAPI)‐diacetate (Molecular Probes). Additional control staining was performed to rule out any nonspecific staining. In each case, sections were blocked with saturating concentrations of appropriate host serum antibodies to eliminate false‐positive staining due to FcR‐mediated nonspecific binding. Staining in the absence of primary antibodies provided additional negative controls. The cell death in salivary gland tissue sections from WT and TRPC1^−/−^ was evaluated by performing the terminal deoxyribonucleotidyl transferase‐mediated triphosphate (dUTP)‐biotin nick end labeling (TUNEL) staining as per manufacturer's instructions (Chemicon International, Temecula, CA). In all the cases, the sections were mounted using FluorSave reagent (Calbiochem, La Jolla, CA) containing 0.3 μM 4′, 6′‐diamidino‐2‐phenylindole (DAPI)‐diacetate (Molecular Probes) and the images were acquired at 40× magnifications using a Nikon Eclipse 80i upright microscope (Nikon Corporation, Tokyo, Japan) with an attached cooled RTke Spot 7.3 three spot color camera (Diagnostic Instruments Inc., Sterling Heights, MI). The images were processed and analyzed using Adobe Photoshop 7.0 software (Adobe, Mountain View, Mountain View, CA).

### 2.9 Statistics

Data analysis was performed using Origin 7.0 (OriginLab) and Graphpad prism 6.0. Statistical comparisons were made using one‐way ANOVA. Experimental values are expressed as means ± SEM or SD. Differences in the mean values were considered to be significant at *P* < 0.05* or <0.01**, respectively. * indicates significance of *P* < 0.05, ** of *P* < 0.01 and *** of *P* < 0.001, respectively.

## 3 RESULTS

### 3.1 SKF induces ER stress and attenuates TRPC1 function/expression thereby inducing cell death

**Figure 1:**
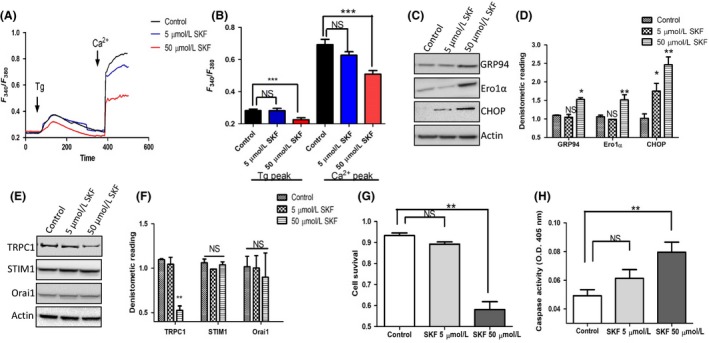
Endoplasmic reticulum (ER) stress and attenuated expression of TRPC1 are induced by SKF pretreatment A, Representative trace showing Tg‐evoked Ca^2+^ entry and Ca^2+^‐evoked [Ca^2+^]_i_ changes in human salivary gland (HSG) cells that are pretreated with 5 μM and 50 μM SKF for 6 h. B, Bar diagram of the fluorescence ratio (340/380) from an average of 80‐100 cells are shown underneath the traces. C, Representative Western blots images showing the expression of ER stress markers in cells pretreated with 5 μM and 50 μM SKF for 12 h. D, Bar diagram showing the densitometer reading (mean ± SD) of expression of the western blot shown in C. Representative Western blots images E, and densitometer reading (mean ± SD) F, showing the expression of calcium entry channels in cells pretreated with 5 μM and 50 μM SKF for 24 h. G, Representative bar diagram showing the cell survival (mean ± SEM) of the cells pretreated with 5 μM and 50 μM SKF for 24 h. H, Bar diagram showing the caspase activity (mean ± SEM) measured in cells pretreated with 5 μM and 50 μM SKF for 24 h. Significance: *, *P* < 0.05; **, *P* < 0.001.

We initially analyzed the role of store‐operated Ca^2+^ entry (SOCE) in modulating salivary gland survival. SKF96345 (SKF) is originally identified as a blocker of Ca^2+^ entry and is widely used as a blocker of transient receptor potential canonical type (TRPC) and ORAI channels. Cells treated with SKF (50uM) attenuated Tg‐mediated Ca^2+^ entry (second peak) in human submandibular gland (HSG) cells (Figure 1A,B), which is consistent with previous published studies.^25^ However, lower doses of SKF only had a minor decrease in Ca^2+^ entry. In addition to Ca^2+^ entry, the ER Ca^2+^ levels were also significantly decreased upon SKF treatment (Figure 1A,B). Consistent with these results, the ER stress markers GRP94, Ero1α, and CHOP were also upregulated when HSG cells were treated with 50 μM SKF for 6 hours, whereas lower concentration of SKF (5 μM) failed to show any changes in the expression of these proteins (Figure 1C,D). Interestingly, prolong treatment of 50 μM SKF (12 hours.) also attenuated TRPC1 channel expression (a 40%‐50% decrease, but no change in the mRNA levels (data not shown)), whereas no effect on either ORAI1 and/or STIM1 expression was observed in HSG cells pretreated with 50 μM SKF for 12 hours. (Figure 1E,F). Moreover, lower concentration of SKF (5 μM) also had no change in TRPC1 protein expression (Figure 1E,F). We next evaluated the consequence of SKF treatment on cell survival and prolonged treatment of higher dose of SKF (50 μM) significantly attenuated the cell survival (Figure 1G). Consistent with the cell survival assays, a dose‐dependent increase in caspase activity was observed in cells treated with SKF, where 50 μM SKF treatment significantly increased the caspase activity (Figure 1 H). Together these results suggest that, in HSG cells, inhibition of SOCE decreases ER Ca^2+^ levels that induces ER stress and promotes cell death.

### 3.2 Tunicamycin and BFA induce ER stress and downregulate TRPC1 expression

**Figure 2:**
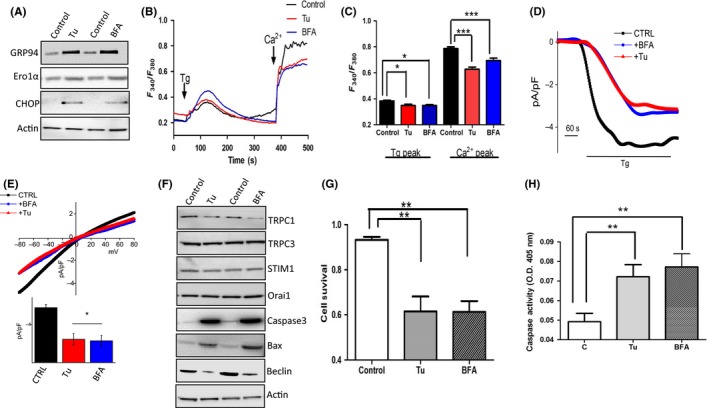
Endoplasmic reticulum (ER) stress and cell death induced by Tunicamycin (Tu) and Brefeldin A (BFA) A, Western blot images showing the expression of ER stress markers in cells pretreated with 10 μM of Tu or BFA, respectively, for 24 h. B, Representative trace showing Tg‐evoked Ca^2+^ entry and ER Ca^2+^ levels in human salivary gland (HSG) cells pretreated with 10 μM Tu or BFA for 6 h. C, Bar diagram of the fluorescence ratio (340/380) from an average of 60‐80 cells are shown. D, Application 1 μM Tg in bath solution induced inward average currents at −80 mV in control and treatment cells. Respectively, IV curves of currents and quantitation (5‐8 recordings) of current intensity at −80 mV are shown in (E). F, Western blot images showing the expression of various calcium channels and autophagy and apoptotic markers in cells pretreated with 10 μM of Tu or BFA, respectively, for 24 h. G, Bar diagram showing the cell survival under various conditions. H, Bar diagram showing the caspase activity measured in cells pretreated with 10 μM Tu and BFA, respectively, for 24 h. Graphs are mean ± SEM, significance: *, *P* < 0.05; **/***, *P* < 0.001.

To further study the relationship between Ca^2+^ entry, ER stress and TRPC1, Tunicamycin (Tu), and Brefeldin A (BFA) which are inhibitors of glycosylation that disturbs protein‐folding machinery in eukaryotic cells causing accumulation of unfolded proteins and inducing ER stress were used. HSG cells pretreated with 10 μM Tu or BFA for 24 hours showed a significant increase in all the ER stress markers evaluated (Figure 2A). Interestingly, cells treated with 10 μM Tu or BFA also attenuated Ca^2+^ entry in HSG cells, along with a significant decrease in ER Ca^2+^ levels (Figure 2B,C). The electrophysiological data further support these results where Tu or BFA treatments significantly decreased Ca^2+^ entry (Figure 2D,E). Importantly, the properties of the Ca^2+^ entry channel in HSG cells were similar to those previously observed with TRPC1 channels 26 which showed a reverse potential around 0 mV and were nonselective (Figure 2D). Importantly, although both Tu and BFA treatment decreased the Ca^2+^ currents, it did not alter the channel properties. These results suggest that TRPC1 channel is the major Ca^2+^ entry channel in these cells, which is affected upon the induction of ER stress. To further establish this, we studied the expression of SOCE components in Tu‐ or BFA‐treated cells. Interestingly, only expression of TRPC1 was downregulated upon Tu or BFA treatment (Figure 2F); whereas no change in the expression of TRPC3, ORAI1, or STIM1 was observed in cells treated with Tu or BFA. Previous studies from our laboratory have shown that TRPC1 plays a vital role in autophagy and cell death,^27^ consistent to that results we also observed a reduction in autophagy markers (LC3A [data not shown] and Beclin). In contrast, an upregulation of apoptotic markers Caspase 3 and Bax was observed in cells pretreated with Tu or BFA. Similarly, addition of ER stress inducers also attenuated the cell survival along with a corresponding increase in caspase activity (Figure 2G,H). Results presented thus far clearly show a correlation between ER stress and TRPC1 expression that could modulate cell survival.

### 3.3 Attenuated TRPC1 expression induces ER stress in HSG cells and enhance the effect induce by Tu and BFA

**Figure 3:**
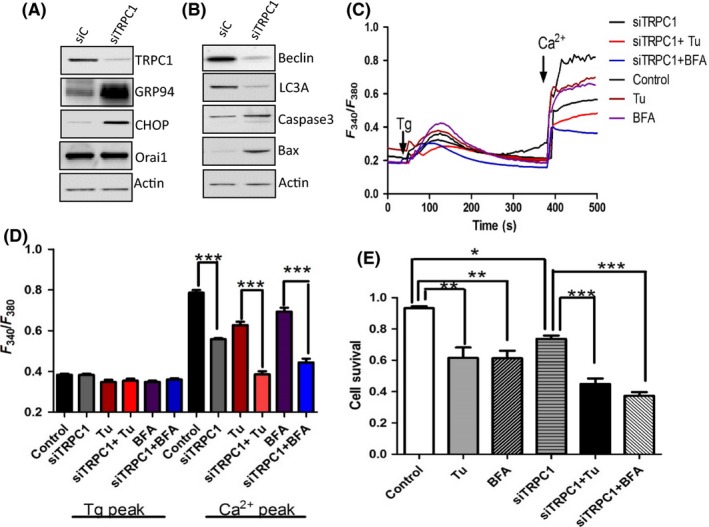
siRNA knockdown of TRPC1 expression in human salivary gland (HSG) cells induces endoplasmic reticulum (ER) stress. A, Western blot images showing the expression of TRPC1, Orai1, and ER stress markers in TRPC1siRNA (siTRPC1) cells and control siRNA (siC), respectively. B, Western blot images showing the expression of autophagy and apoptotic markers in TRPC1siRNA (siTRPC1) cells and control siRNA (siC), respectively. C, Representative trace showing changes in [Ca^2+^]_i_ on cells pretreated with 10 μM Tunicamycin (Tu) or Brefeldin A (BFA) for 6 h. D, Bar diagram of the fluorescence ratio (340/380) from an average of 50‐60 cells are shown. E, Bar diagram showing the cell survival of the cells pretreated with 10 μM Tu and BFA, respectively, for 24 h in siRNA control and TRPC1 knockdown HSG cells. Graphs are mean ± SEM, significance: *, *P* < 0.05; **/***, *P* < 0.001.

**Figure 4:**
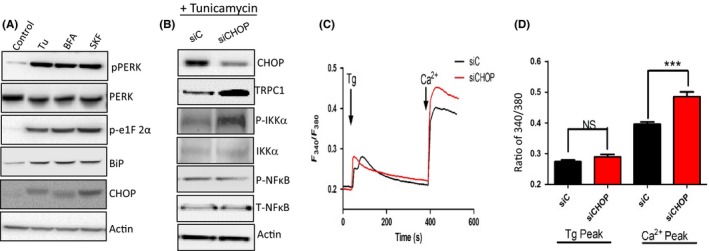
SKF, Tunicamycin (Tu), and Brefeldin A (BFA) induce endoplasmic reticulum (ER) stress mainly through PERK phosphorylation and C/EBP homologous protein (CHOP) upregulation. A, Western blot images showing the expression of various proteins as labeled in cells pretreated with 10 μM of Tu or BFA or 50 μm SKF, respectively, for 24 h. B, Western blot showing expression of various proteins in control siRNA (siC) and CHOP knockdown cells (siCHOP) along with Tu treatment. C, Representative trace showing Tg‐evoked Ca^2+^ entry and Ca^2+^ entry in control siRNA (siC) and siCHOP‐treated human salivary gland (HSG) cells. D, Bar diagram of the fluorescence ratio (340/380) from an average of 50‐60 cells is shown. Graphs are mean ± SEM, *** indicates significance (*P* < 0.01).

Thus, to establish the significance of TRPC1 in the induction of ER stress, we inhibited TRPC1 expression in these cells by using the siRNA approach. Addition of TRPC1siRNA showed a 70% knockdown of TRPC1 protein when compared with control siRNA (Figure 3A). Moreover, TRPC1 silencing also upregulated the expression of ER stress markers, mainly GRP94 and CHOP (Figure 3A). We next evaluated the effect of TRPC1 silencing on autophagy and cell survival. Similar to ER stress markers, apoptotic markers mainly caspase 3 and Bax were upregulated, whereas a decrease in both LC3A and Beclin expression was observed in TRPC1‐silenced cells (Figure 3B). Inhibition of TRPC1 expression also inhibited Tg‐mediated Ca^2+^ entry and silencing of TRPC1 in HSG cells enhanced the effect of Tu and BFA, which showed a further decrease in Ca^2+^ entry in cells treated with Tu or BFA along with expressing TRPC1siRNA (Figure 4C,D). Consequently, the cell survival was also attenuated in cells treated with siTRPC1 and silencing of TRPC1 further enhanced the Tu‐ and BFA‐induced cell death (Figure 3E). Together results presented thus far suggest that loss of TRPC1 expression and function dictates ER stress that induces loss of salivary gland cells.

### 3.4 SKF, Tu, and BFA induce ER stress in HSG mainly through perk phosphorylation and CHOP upregulation that modulates TRPC1 expression

To understand the mechanism as how ER stress is further propagated, we next evaluated all the downstream pathways that are activated in ER stress conditions. Three major signaling pathways (pIRE1, pPERK, and ATF6) have been identified that coordinate the cellular response to unfolded proteins. HSG cells treated with either Tu or BFA showed an increase induction of the phosphorylation of PERK (Figure 4A), whereas no change in either pIRE1 or activation of transcription factor ATF6 was observed (data not shown). Although early activation of PERK is protective under stress conditions, it also leads to the induction of CHOP which is an important element to switch from pro‐survival to pro‐death signaling.^14^ Interestingly, cells treated with SKF (50 µM), which blocks Ca^2+^ entry also increased the phosphorylation of PERK (Figure 4A). Similarly, the expression of downstream targets of pPERK, like BiP, phosphor—e1F2 alpha and CHOP expression were also increased in cells treated with either SKF, Tu, or BFA (Figure 4A). One of the puzzling results in our studies was why expression of TRPC1 was decreased in ER stress conditions and also ER stress‐mediated cell death has been shown to be dependent on CHOP expression 16, 28; thus, we silenced CHOP and evaluated its effect on TRPC1 expression and Ca^2+^ entry. Inhibition of CHOP expression using siRNA (siCHOP), significantly decreased Tu‐induced increase in CHOP expression (Figure 4B). Importantly, silencing of CHOP showed a significant increase in TRPC1 expression (Figure 4B). We have previously shown that activation of NFkB is important for TRPC1 expression.^27^, ^28^ Surprisingly, no change in p‐NFκB expression was observed in CHOP‐silenced cells (Figure 4B). In contrast, p‐IKKα that phosphorylates IkBα thereby leading to its degradation was increased. Loss of IkBα allows the movement of NFkB to the nucleus, which could modulate gene expression including TRPC1. Consistent with these results, silencing of CHOP also increased Ca^2+^ entry, where significant increase in Tg‐mediated Ca^2+^ entry was observed in cells treated with siCHOP (Figure 4C,D). These results suggest that CHOP modulates TRPC1 expression that produces a prolonged effect thereby leading to cell death.

### 3.5 TRPC1 knockout mice show increase ER stress and immune infiltration in salivary gland cells

**Figure 5:**
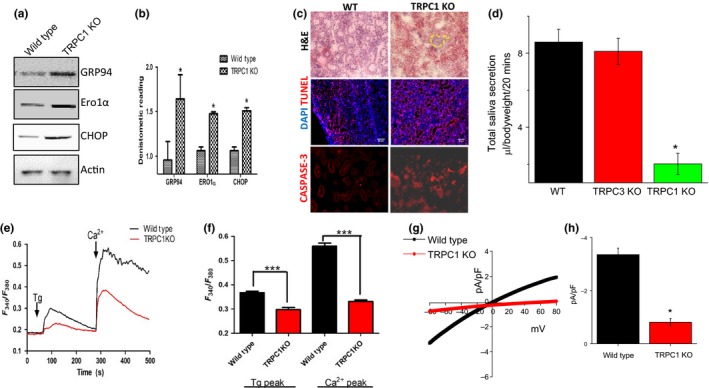
Attenuated production of Saliva in TRPC1 knockout mice relates to endoplasmic reticulum (ER) stress. (A,B), Western blot images and bar diagram of the densitometer reading (mean ± SD) showing the expression of various ER stress markers in TRPC1 knockout mice salivary gland tissue samples. C, H&E‐stained SMG sections showing immune infiltration as well as representative confocal images showing expression of caspase 3, AQP5, and Tunnel staining in control and TRPC1 knockout SMGs. DAPI was used for nuclear staining. D, Total saliva secretion (mean ± SEM) in WT, TRPC1 KO, and TRPC3 KO mice. E, Representative trace showing Tg‐evoked changes in Ca^2+^ in wild type and TRPC1 KO mice salivary gland cells. F, Bar diagram (mean ± SEM) of the fluorescence ratio (340/380) from an average of 50‐60 cells is shown. G, IV curves of currents induced by Tg in WT and TRPC1 KO cells and quantitation (5‐8 recordings) of current intensity at –80 mV. (H),Combined data are shown as bar graph. Significance: *, *P* < 0.05; ***, *P* < 0.001.

Data presented thus far indicate that loss of TRPC1 in salivary gland cells lead to ER stress that could account for the loss of salivary gland cells. Thus, to understand the physiological relevance of the TRPC1 channel in ER stress and salivary gland function, we used control and TRPC1 knockout mice.^24^, ^29^ Mice‐lacking TRPC1 expression showed a significant increase in ER stress markers and expression of GRP94, Ero1α, and CHOP were significantly increased in TRPC1 knockout mice, when compared with age‐matched wild‐type control mice (Figure 5A,B). Consistent to the results presented above increased caspase 3 expression as well as increased Tunnel staining was observed in TRPC1 knockout mice. Importantly, H&E staining also confirmed increase in immune infiltration in TRPC1 knockout mice when compared with wild‐type control mice (Figure 5C), which were mostly dendritic cells that are essential for T‐cell response (data not shown). Similarly, TRPC1 knockout mice, but not TRPC3 knockout mice, showed a reduction in saliva secretion when compared with control mice (Figure 5D). To fully establish that ER stress is due to the loss of Ca^2+^ homeostasis, both ER and cytosolic Ca^2+^ levels were evaluated in salivary gland cells of control and TRPC1 knockout mice. Importantly, a significant decrease in Ca^2+^ entry was observed in TRPC1 knockout mice along with a similar reduction in ER Ca^2+^ levels, when compared with acinar cells isolated from mice submandibular glands (Figure 5E,F). Similarly, Tg‐induced Ca^2+^ currents were also attenuated in TRPC1 knockout mice (Figure 5G,H). Together these results suggest that loss of Ca^2+^ entry via the TRPC1 channel decreases ER Ca^2+^ levels that induce ER stress followed by increased loss of salivary gland cells and immune infiltration.

### 3.6 Overexpression of TRPC1 rescues the Tu‐ and BFA‐mediated attenuation of SOCE and cell death

**Figure 6:**
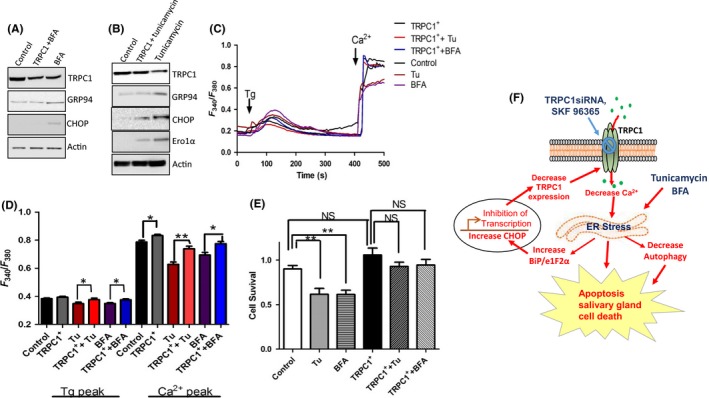
Overexpression of TRPC1 rescues the effect of Tunicamycin (Tu) and Brefeldin A (BFA). (A,B), Respective western blots showing the expression of various endoplasmic reticulum (ER) stress markers in TRPC1 overexpressed cells pretreated with Tu and BFA. C, Representative trace showing Tg‐evoked Ca^2+^ entry and Ca^2+^‐evoked [Ca^2+^]_i_ changes in various conditions as labeled in the figure. D, Bar diagram of the fluorescence ratio (340/380) from an average of 40‐60 cells. E, Bar diagram showing the cell survival of the cells pretreated with 10 μM Tu or BFA, respectively, for 24 h in TRPC1 overexpressed human salivary gland (HSG) cells. F, Schematic model showing the role of TRPC1 channels in ER stress and salivary gland cell death. Graphs are mean ± SEM, significance: *, *P* < 0.05; **, *P* < 0.001.

Data presented thus far clearly show the importance of TRPC1 in modulating ER stress; thus, we next evaluated if overexpression of TRPC1 could inhibit ER stress and induce cell survival. Importantly, HSG cells overexpressing full‐length TRPC1 showed inhibition of ER stress and expression of ER stress markers were decreased in cells treated with Tu or BFA in cells that overexpress full‐length TRPC1 (Figure 6A,B). Consistent with these results, overexpression of TRPC1 restored Ca^2+^ entry which was decreased by the addition of either Tu or BFA (Figure 6C,D). Furthermore, ER Ca^2+^ levels were also restored in cells treated with Tu or BFA that also overexpress TRPC1. Finally, the cell survival assays also showed a significant reduction in Tu‐ and BFA‐mediated cell death in cells that overexpress TRPC1 (Figure 6E). Together these results clearly show that loss of TRPC1 decreases Ca^2+^ homeostasis that is essential for inhibiting ER stress and induction of apoptosis.

## 4 DISCUSSION

Disturbances in ER and cytosolic Ca^2+^ homeostasis have been associated with many diseases including salivary glands.^30^ Disruption of ER Ca^2+^ homeostasis induces the UPR, which is a pro‐survival defense mechanism that prevents further accumulation of newly synthesized proteins in the ER in order to reduce further burden to the ER However, prolonged UPR activation occurs when physiological mechanisms fail to restore normal ER function, thereby causing ER stress and cell death,^31^ but the mechanism as how ER stress is initiated that is further prolonged to cause cell death is not well known. Our studies provide evidence that inhibition of Ca^2+^ entry is one of the initiating factors that could lead to ER stress. Furthermore, our results also show that TRPC1 is the major Ca^2+^ entry channel in salivary gland cells and loss of TRPC1 function lead to initiation of ER stress. Importantly, besides TRPC1, other TRPC channels and ORAI1 has been shown to play a role in modulating Ca^2+^ entry as well as in maintaining cellular functions 16, 28; however, surprisingly no change in ORAI1 expression or other TRPC channels was observed in the presence of stressors that induce ER stress. ER stress‐inducing agents (Tu and BFA) lead to Ca^2+^ depletion during the early phase that promoted loss of ER Ca^2+^ levels and the onset of ER stress. Furthermore, activation of CHOP leads to a decrease in TRPC1 expression which together further attenuated autophagy and increases apoptosis which results in cell death; a schematic diagram is represented in Figure 6F to illustrate the results.

Tu and BFA have been previously used to induce ER stress.^32^ BFA, a macrocyclic lactone antibiotic synthesized from palmitate by a variety of fungi, induces endoplasmic reticulum (ER) and Golgi stress via the inhibition of ADP ribosylation factor (ARF), resulting in the reduction in coatomer protein assembly and the disruption of ER‐Golgi vesicular transport.^33^ Pretreatment of cells with ER stress inducers attenuated TRPC1‐mediated Ca^2+^ entry in salivary gland cells. Ca^2+^ entry through TRPC1 channels not only ensures optimal refilling of the ER but also increase cytosolic Ca^2+^ levels essential for the activation of various kinases.^16^ Importantly, SOC‐mediated Ca^2+^ entry decreased in the presence of ER stress‐inducing agents, and since only TRPC1 expression was decreased, we infer that the loss of endogenous SOC‐mediated Ca^2+^ entry was due to the loss of TRPC1. There is a strong possibility that ER stress‐inducing agents could directly inhibit TRPC1 channel activity; however, more research is needed to explore this concept and a more detail timeline is needed to further explore the exact sequence of these events. Nonetheless, these results are important as silencing of TRPC1 mimicked similar ER stress response that was observed with ER stress agents. Importantly, addition of Tu or BFA in TRPC1‐silenced cells showed a further decrease in calcium entry and cell survival. In contrast, restoration of TRPC1 expression was able to blunt the effects of Tu and BFA and inhibited ER stress and prevented the subsequent loss of salivary gland cells, suggesting that TRPC1 is the major calcium channel in salivary gland that could inhibit ER stress response. Consistent with these results, the TRPC1 knockout mice also showed increase in ER stress markers, along with a decrease in calcium homeostasis as well as cell death markers were also increased in TRPC1 knockout mice. Calcium has been shown to modulate apoptosis 2, 7, 34 which suggest that loss of TRPC1‐mediated calcium entry could be the main trigger. Importantly, increased immune infiltration was also observed in TRPC1 knockout mice suggesting that ER stress‐induced loss of salivary tissues could be a factor in autoimmune diseases such as SS that effects salivary glands and is characterized by increase in immune cells.^35^ However, further research is needed to clearly establish this link and to show if loss of calcium entry is a factor in modulating SS and to clarify the types of immune cells infiltrating in TRPC1 KO mice.

The major protective and compensatory mechanism during ER stress is the UPR, which leads to translational attenuation and selective upregulation of a number of bZip transcription factors.^9^ UPR serves multiple functions, including the assistance of protein folding via the upregulated ER protein chaperones and the enhanced degradation of misfolded proteins via the upregulation of molecules involved in the ER‐associated degradation pathway.^14^ Dissociation of GRP78 from PERK initiates the dimerization and autophosphorylation of the kinase and generates active PERK CHOP, which is an important mediator of ER stress‐induced apoptosis.^9^, ^34^, ^36^ The transcription factor CHOP, a multifunctional transcription factor in the ER stress response, whose induction strongly depends on ATF4, is well known to promote apoptotic cell death.^34^, ^37^ In the present study, we show that silencing of CHOP resulted in attenuated expression of TRPC1 in the cells which could result in decreased SOCE. Moreover, the CHOP and Ero1α expression and activity were both required for triggering IP_3_R‐mediated ER Ca^2+^ release,^38^ a critical step in ER stress‐induced apoptosis,^34^ indicating that under stress conditions, Ero1α can in fact regulate ER Ca^2+^ content. Another important observation was that inhibition of CHOP expression leads to increase in TRPC1 expression. Although the exact mechanism is not clear, loss of CHOP could have facilitated the movement of NFkB to the nucleus by the degradation of IkBα as increased phosphorylation of IKK was observed. These results are consistent with previous studies where NFkB has been shown to bind to TRPC1 promoter and were essential for its expression.^25^ Overall, our results not only show the role of TRPC1 in ER stress in salivary gland cells and tissues but also provide the mechanism as how ER stress is induced as well as have identified TRPC1 as a key factor that could prevent ER stress in salivary gland cells. These results are important as they could identify novel mechanism that is altered in salivary gland dysfunction such as SS.

## CONFLICT OF INTEREST

The authors declare no conflict of interest.

## AUTHOR CONTRIBUTIONS

P.S. performed the calcium experiments, cell survival, caspase activity, western blots and drafted the manuscript. Y.S. performed the electrophysiological studies, saliva secretion and was involved in the interpretation of data. F.Q.Z. carried out the immunohistochemistry and N.C. performed some of the western blots and their quantification. B.M. participated in the design and interpretation of the data. B.S. conceived the study, design experiments, performed analysis and interpretation of data, coordinated and helped to draft the manuscript. All authors read and approved the final manuscript.
