# Supplementary material for: TRPC1 expression and function inhibit ER stress and cell death in salivary gland cells
Source: FASEB Bioadv. 2018 Nov 16;1(1):40–50. doi: 10.1096/fba.1021 (PMC6524637; doi:10.1096/fba.1021)
Supplement: Supplementary file 1 [file FBA2-1-40-s001.docx]

**Supplementary Table S1**

| Antibody | Company | Catalog Number | Dilution Used |
| --- | --- | --- | --- |
| 1. TRPC1 | Alomone labs | ACC-010 | 1:500 |
| 1. TRPC3 | Alomone labs | ACC-016 | 1:500 |
| 1. TRPC4 | Alomone labs | ACC-0119 | 1:500 |
| 1. TRPC5 | Alomone labs | ACC-020 | 1:500 |
| 1. Actin | Cell Signaling Technology | 4970 | 1:2000 |
| 1. STIM1 | Cell Signaling Technology | 5668 | 1:1000 |
| 1. Orai1 | Alomone labs | ACC-060 | 1:1000 |
| 1. TH | Cell Signaling Technology | 2792 | 1:1000 |
| 1. LC3A | Cell Signaling Technology | 4599 | 1:1000 |
| 1. Beclin | Cell Signaling Technology | 3495 | 1:1000 |
| 1. p62 | Cell Signaling Technology | 8025 | 1:1000 |
| 1. Bax | Cell Signaling Technology | 5023 | 1:1000 |
| 1. Caspase 3 | Cell Signaling Technology | 9662 | 1:1000 |
| 1. AMPK | Cell Signaling Technology | 5831 | 1:1000 |
| 1. pAMPK | Cell Signaling Technology | 2535 | 1:1000 |
| 1. ATG7 | Cell Signaling Technology | 8558 | 1:1000 |
| 1. CytoC | Cell Signaling Technology | 11940 | 1:1000 |
| 1. pPKCα | Cell Signaling Technology | 9375 | 1:1000 |
| 1. PKCα | Cell Signaling Technology | 59754 | 1:1000 |
| 1. AKT(Thr308) | Cell Signaling Technology | 13038 | 1:1000 |
| 1. AKT(Ser473) | Cell Signaling Technology | 4060 | 1:1000 |
| 1. AKT | Cell Signaling Technology | 9272 | 1:1000 |
| 1. pNFκB | Cell Signaling Technology | 3033 | 1:1000 |
| 1. NFκB | Cell Signaling Technology | 8242 | 1:1000 |
| 25. LC3A/B. | Cell Signaling Technology | 12741 | 1;1000 |
